# Genome‐wide diversity evaluation and core germplasm extraction in ex situ conservation: A case of golden *Camellia tunghinensis*

**DOI:** 10.1111/eva.13584

**Published:** 2023-08-19

**Authors:** Xianliang Zhu, Rong Zou, Huizhen Qin, Shengfeng Chai, Jianmin Tang, Yingying Li, Xiao Wei

**Affiliations:** ^1^ Guangxi Key Laboratory of Plant Functional Phytochemicals and Sustainable Utilization Guangxi Institute of Botany, Guangxi Zhuang Autonomous Region and Chinese Academy of Sciences Guilin China; ^2^ Institute of Forestry Economic Science, Guangdong Academy of Forestry Guangzhou China

**Keywords:** *Chrysantha*, conservation genomics, core collection, genetic differentiation, population structure, SNP

## Abstract

Whether ex situ populations constructed in the limited nursery resources of botanical gardens can preserve enough genetic diversity of endangered plants in the wild remains uncertain. Here, a case study was conducted with *Camellia tunghinensis*, which is one of the species with the lowest natural distribution area in the sect. *Chrysantha* (golden camellia) of the family Theaceae. We investigated the genetic diversity and population structure of 229 samples from wild and ex situ populations using genotyping by sequencing (GBS). Core germplasm was constructed from these samples. The results showed that wild *C. tunghinensis* exhibited high genetic diversity, with observed heterozygosity of 0.257–0.293 and expected heterozygosity of 0.247–0.262. Compared with wild populations, the genetic diversity of ex situ populations established by transplanting wild seedlings was close to or even higher. However, the genetic diversity of those established by seed or cuttings of a few superior trees was lower. The Admixture analysis revealed that the structure of the ex situ populations derived from seeds and cuttings was relatively simple compared with the ex situ populations derived from transplanted wild seedlings and wild populations. These results suggested that direct transplanting of wild seedlings was more conducive to preserving the genetic diversity of endangered plants in the wild. In addition, wild populations demonstrated a small differentiation (mean *F*_ST_ = 0.044) among themselves, possibly due to long‐term and frequent gene flow between the wild populations. In contrast, moderate differentiation (mean *F*_ST_ > 0.05) was detected among ex situ populations and between ex situ and wild populations. This may be the combined result of the absence of gene flow pathways and strong selection pressure in various ex situ environments. Finally, 77 core germplasms were extracted from 229, likely representing the genetic diversity of *C. tunghinensis*. This study provides future strategies for the ex situ conservation and management of the golden camellia species and other rare and endangered plants.

## 1 INTRODUCTION

Ex situ conservation is a specific protection and management strategy to preserve the genetic diversity of species. It refers to the off‐site management of species whose survival and reproduction in their native habitat are seriously threatened (Cibrian‐Jaramillo et al., 2013; Fay, 2010). Due to global climate change and frequent human interference, an increasing number of wild plant populations continue to shrink and face pressures for their survival. Ex situ conservation has become one of the most significant means to save these endangered plants. However, botanical gardens or germplasm nurseries established via ex situ conservation are fundamentally an artificially created collection of small isolated populations (Ensslin et al., 2015). Therefore, endangered plants in ex situ conservation have the same risk of decline in genetic diversity or extinction as those in small wild populations. In addition, many ex situ conservation programs are characterized by incomplete sampling, duplicate introductions, unclear resources, and poor planting conditions during the collection and introduction of germplasm resources (Kang et al., 2005; Wei & Jiang, 2021). This leads to uncertainty in terms of whether these ex situ populations formed with limited nursery resources are comparable in genetic diversity to wild populations.

Information on the genetic diversity and population structure of ex situ populations is most important for assessing the success of ex situ conservation (Cibrian‐Jaramillo et al., 2013; Clugston et al., 2022; Mueller et al., 2022). If the genetic diversity of ex situ populations is consistent with, similar to, or higher than that of wild populations, the ex situ conservation programs are considered to be preliminarily successful, and if not, further improvements are required. Many studies have demonstrated that genetic diversity in species is influenced by multiple factors, including gene flow (Feliciano et al., 2022; Su, Richardson, et al., 2017), selection (Luo et al., 2022; Niu et al., 2019), evolutionary history (Guerra‐Garcia et al., 2022; Roy et al., 2017), and the level of inbreeding (Dicks et al., 2023). For example, Feliciano et al. (2022) found low population genetic diversity associated with restricted gene flow in the endemic and endangered species *Portulaca hatschbachii* in Brazil. A large‐scale genomic analysis of walnuts (*Juglans regia*) revealed the effect of selection on genetic diversity, and they found slower rates of linkage disequilibrium (LD) decay and lower levels of nucleotide diversity in domesticated cultivars and landraces compared with wild walnuts (Luo et al., 2022). Thus, in addition to understanding the genetic diversity of the endangered plants, it is important to explore the factors shaping their genetic diversity. Furthermore, the lack of such information may also pose a range of genetic risks to the endangered plants being protected, including genetic drift, decline due to inbreeding, and reduced or absent adaptability (Havens et al., 2004; Shemesh et al., 2018). These risks may be more pronounced specifically when considering wild reintroduction following ex situ conservation (Kang et al., 2005).

*Camellia* sect. *Chrysantha* Chang (golden camellia) is a section of evergreen shrubs or small trees and is the only section with yellow flowers in the family Theaceae, also known as the “queen of camellias” and “giant panda of the plant world” (Liu et al., 2022). In recent decades, due to human overexploitation, wild golden camellias have become endangered and have been included on the List of National Key Protected Wild Plants in China (State Forestry Administration of P. R. China & Ministry of Agriculture and Rural Affairs of P. R. China, 2021). In order to effectively conserve golden camellia species, ex situ conservation programs were begun as early as 1980, and many researchers, including our colleagues, have completed resource surveys, germplasm collections, and introduction and domestication of golden camellia species (Wei et al., 2005). Although achieving a life history of “seed to seed” can signal initial success in ex situ populations (He, 2002), these results should also be combined with an evaluation of genetic diversity. Only a few studies have investigated the genetic diversity of ex situ populations of *Camellia nitidissima* based on few intersimple sequence repeat (ISSR) markers (Wei et al., 2005, 2008) without the support of genome‐wide markers. In contrast, most research has focused on the genetic diversity of wild populations of golden camellia species, such as *C. nitidissima* (Tang et al., 2006), *Camellia flavida* (Wei et al., 2017), and *Camellia chrysanthoides* (Chen et al., 2019).

**FIGURE 1:**
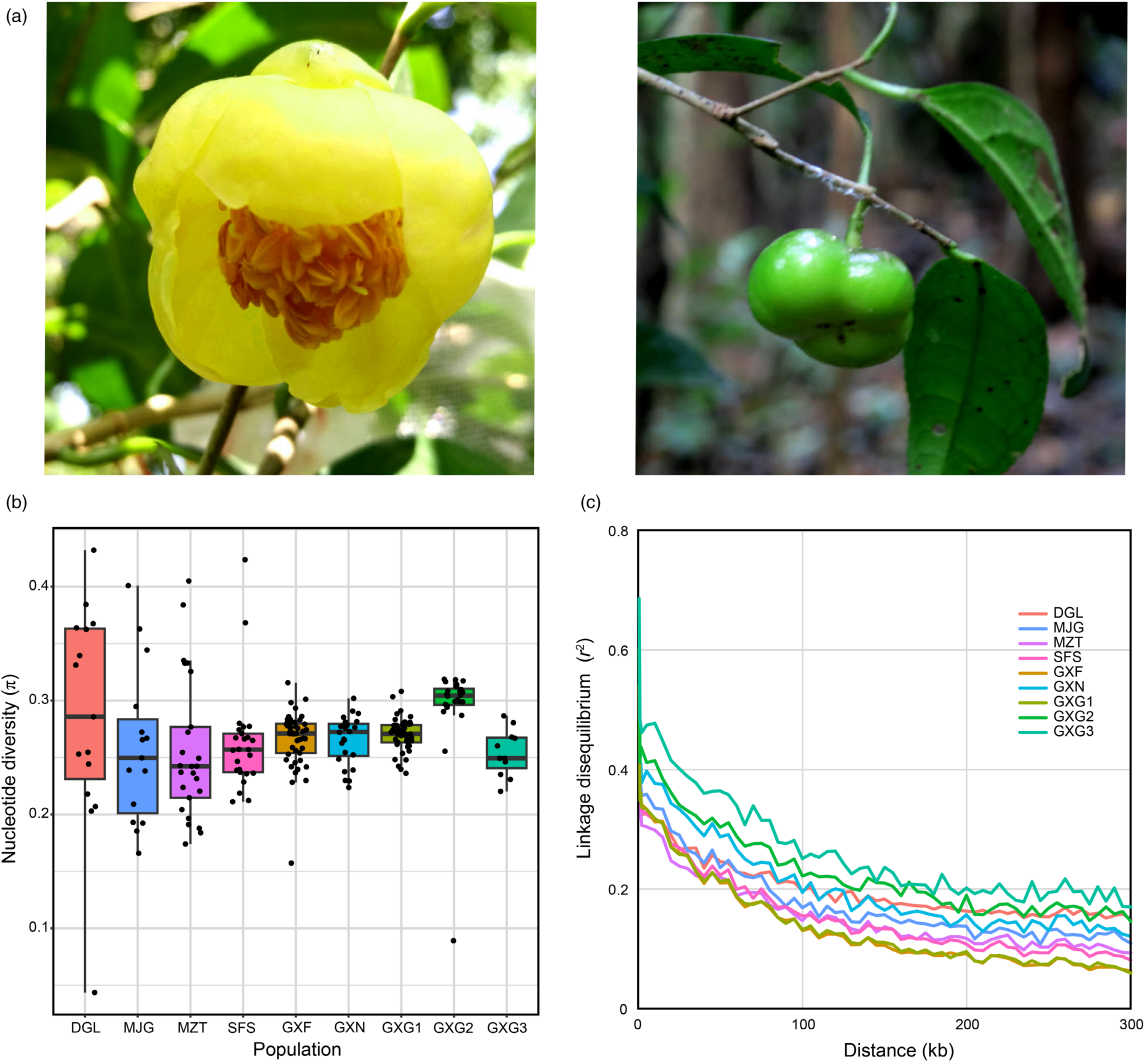
Morphological characterization and genetic diversity analysis of *C. tunghinensis*. (a) Demonstrates the flower (left) and fruit (right) of *C. tunghinensis*. (b) Shows the distribution of nucleotide diversity for each individual (each point) in each population. (c) Shows the LD decay for each population, with flatter lines indicating slower decay.

*Camellia tunghinensis* is one of the golden camellia species with the smallest natural distribution and is currently restricted to Fangchenggang, Guangxi, China (Nong et al., 2010). With its dense branches, lush flowers, and beautiful tree shape, *C. tunghinensis* has significant ornamental value and is also an excellent germplasm resource for breeding new *Camellia* varieties (Figure 1a; Qin et al., 2022). Three public organizations (see Section 2.1 below) have already implemented ex situ conservation programs to conserve *C. tunghinensis*. To date, however, no genetic diversity evaluation has been conducted for these ex situ populations. Furthermore, the degree of genetic differentiation among these populations remains unclear.

The rapid development of high‐throughput sequencing has expanded our understanding of population genetic diversity and differentiation of endangered species in an unprecedented way, allowing efficient identification of core germplasms (Chen, Zhang, et al., 2022; Lin et al., 2021; Su, Wang, et al., 2017). Thus, this study aimed to develop large‐scale genome‐wide SNP markers via high‐throughput sequencing to assess the genetic diversity, population structure, and differentiation of wild and ex situ populations of *C. tunghinensis* and to enhance conservation by constructing a core germplasm resource. The results of this study provide strategies for the ex situ conservation, management, and utilization of the golden camellia species and other endangered woody perennials.

## 2 MATERIALS AND METHODS

### 2.1 Plant materials

At present, wild *C. tunghinensis* is only found in an area of less than 100 hm^2^ on the western slopes of the core area of Shangyue in the Golden Camellia National Nature Reserve of Fangchenggang, Guangxi, China, with an actual area of 52 hm^2^ (Nong et al., 2010). There are four naturally occurring wild populations: Dagoulong (DGL), Mijigou (MJG), Mizaitian (MZT), and Sifangshan (SFS; Table 1). The ex situ conservation programs of five ex situ populations of *C. tunghinensis* were implemented by three public organizations, including the Guangxi Institute of Botany, the Golden Camellia National Nature Reserve of Fangchenggang, and the Nanning Golden Camellia Park (Table 1). Of these, the germplasm of the Guangxi Fangchenggang (GXF), Guangxi Nanning (GXN), and Guangxi Guilin 1 (GXG1) populations all originated from transplanted wild seedlings (planted between 1985 and 1990). The Guangxi Guilin 2 (GXG2) population originated from the seeds of an elite wild tree (planted around 2000). The Guangxi Guilin 3 (GXG3) population originated from the cuttings of another elite wild tree (planted in 2015). The leaf samples were randomly collected from 80 adult trees in four wild populations and all 149 adult trees in the five ex situ populations (Table 1). Notably, the 149 adult trees in the five ex situ populations were not derived from propagated ex situ collections.

**TABLE 1:** Sampling information and genetic diversity analysis.

Population	DGL	MJG	MZT	SFS	GXF	GXN	GXG1	GXG2	GXG3
Type	Wild population				Ex situ population				
Longitude	108°5′37″	108°5′27″	108°5′57″	108°5′55″	108°6′41″	108°21′27″	110°18′2”		
Latitude	21°44′49″	21°44′53″	21°45′6″	21°45′4″	21°44′48″	22°49′10″	25°4′13″		
Habitat	Near valley	Near valley	Near valley	Near valley and slopes	Flat land under aniseed forest	Flat land	Flat land under pine forest		
No. of adult trees	<50	<50	<100	<100	45	24	45	24	11
Sample	15	15	25	25	45	24	45	24	11
*H*_E_	0.259	0.262	0.247	0.258	0.277	0.238	0.275	0.243	0.211
*H*_O_	0.293	0.261	0.257	0.262	0.266	0.265	0.270	0.297	0.253
π	0.268	0.271	0.252	0.263	0.280	0.244	0.278	0.248	0.221
*F*_IS_	−0.018	0.035	0.004	0.013	0.046	−0.050	0.029	−0.071	−0.073
Tajima'D	0.686	0.422	0.496	0.472	0.682	0.556	0.655	0.669	0.483

Abbreviations: *F*_IS_, inbreeding coefficient; *H*_E_, expected heterozygosity; *H*_O_, observed heterozygosity; π, nucleotide diversity.

### 2.2 GBS library construction and sequencing

The genomic DNA of 229 samples was extracted using the E.Z.N.A. Tissue DNA kit (Omega Bio‐Tek, USA) according to the manufacturer's instructions. The DNA quality was assessed using 1% agarose gel electrophoresis and a Nanodrop2000 (ThermoFisher, USA) spectrophotometer. The DNA was quantified using Qubit3.0 (ThermoFisher), ensuring that each sample met the following criteria: total mass >3 μg, concentration > 30 ng/μl, and OD260/OD280 = 1.80–2.00. The quality‐checked DNA was double‐digested using an enzyme combination of *EcoRI* and *NlaIII*, as described by Wang et al. (2017). Next, the barcode adapters were ligated, followed by fragment size selection and PCR amplification to obtain ddGBS libraries with a target length of 400–600 bp. The constructed libraries were sent for 150‐bp paired‐end sequencing on the Illumina Novaseq 6000 platform.

### 2.3 SNP calling

Quality assessment of the raw data was performed using FastQC (Andrews, 2010). Adapter sequences, cut sites (the first six base pairs of reads), and low‐quality reads (Q20 <20, length <36 bp) in the raw sequences were filtered with Trimmomatic v0.36 (Bolger et al., 2014) using default parameters. The clean reads were then mapped to the *Camellia sinensis* reference genome (version GCA_013676235.10, https://www.ncbi.nlm.nih.gov/) using BWA v0.7.12 (H. Li & Durbin, 2009). The SNPs were called using GATK v4.1.2.0 (McKenna et al., 2010) with default parameters. VCFtools v0.1.11 (Danecek et al., 2011) was used to remove variants with more than two alleles, Phred scores below 30, and population deletion rates above 5% (i.e., detected in less than 95% of individuals) to obtain high‐quality SNP data for downstream analysis.

### 2.4 Analysis of population genetics

Based on filtered high‐quality SNP data, genetic diversity parameters including observed heterozygosity (*H*_O_), expected heterozygosity (*H*_E_), nucleotide diversity (π), and inbred lineage coefficient (*F*_IS_) were calculated by Stacks v2.59 (Rochette et al., 2019). The Tajima'D value was calculated for all loci in 3000‐bp nonoverlapping windows across the genome of each population using VCFtools. Bayesian clustering was performed on all individuals using Admixture v1.3.0 (Alexander et al., 2009) with clustering values (*K*) set from 1 to 20. The optimal *K* value was determined based on the minimum cross‐validation (CV) error (Wang et al., 2022). Principal component analysis (PCA) was performed using Plink v1.07 (Chang et al., 2015). A phylogenetic tree was constructed for the 229 individuals using the maximum likelihood (ML) method in FastTree v2.1.11 (Price et al., 2010) software. The “‐nt ‐gtr” parameter was used to specify the nucleotide alignment model. The tree was visualized using the online tool Phylogenetic Tree View by ImageGP (Chen, Liu, & Huang, 2022). The pattern of LD decay for each population was estimated using PopLDdecay (Zhang et al., 2019).

### 2.5 Genetic differentiation and gene flow analysis

Genetic differentiation coefficients (*F*_ST_) between the nine populations were calculated using VCFtools. As the ex situ populations were artificially constructed and it was known that there were gene flow barriers between them, three ex situ populations were excluded from the subsequent gene flow analysis. Treemix v1.12 (Pickrell & Pritchard, 2012), a method used to detect historical migration events (i.e., historical gene flow), was used to detect gene flow between the wild populations. Since the Treemix analysis relies on a missing‐free dataset, we regenerated a missing‐free dataset of 80 wild individuals, including a total of 25,185 SNPs. The migration events (m) were assumed to be between 0 and 5. The Evanno algorithm in the R package “OptM” (Fitak, 2021) was then used to determine the optimal migration model.

### 2.6 Core germplasm construction

For efficient conservation and utilization of the germplasm resources of *C. tunghinensis*, the core germplasm was constructed using CoreHunter3 (De Beukelaer et al., 2018). Briefly, CoreHunter3 can extract the optimal core set by simultaneously maximizing allelic richness and genetic dissimilarity. The method is recognized as one of the most efficient and popular core set algorithms available (De Beukelaer et al., 2018). First, the genetic distance matrix of 229 individuals in the wild and ex situ populations was calculated using Plink. The sampling intensity of previous core germplasms of woody plants usually ranges between 30% and 45% (Duan et al., 2017; Lv et al., 2020; Wang et al., 2016; Zhao et al., 2022). In the *Camellia* genus, Zhao et al. (2022) determined a sampling intensity of 33.6% for the core germplasm representing maximum genetic diversity based on the pruned edge length of the initial tree length and the sphericity index, allowing the constructed core germplasm to be used for further breeding studies. Therefore, with reference to this study (Zhao et al., 2022), the sampling intensity was set to 33.6% and the core germplasm was extracted in CoreHunter3 based on the genetic distance matrix. Finally, the genetic diversity of the core germplasm was calculated using the abovementioned method (see Section 2.4).

## 3 RESULTS

### 3.1 SNP calling based on the reference genome

A total of 5,518,804,554 raw reads (810 Gb) were generated from 229 *C. tunghinensis* samples. Following quality control, an average of 22.9 million clean reads were obtained per sample (Table S1). The clean reads of each sample demonstrated a high mapping rate in the reference genome with an average of 97.36%. GATK was used to detect a total of 12,823,534 raw SNPs. Further screening of the raw SNPs resulted in a total of 134,613 high‐quality SNPs, evenly distributed across the reference genome with an average of one SNP per 23.1 kb (Figure S1).

### 3.2 Genetic diversity

The genetic diversity of *C. tunghinensis* was calculated using 134,613 SNPs. Among the nine populations, GXG2 displayed the highest *H*_O_ (0.297) while the lowest was observed in GXG3 (0.253; Table 1). The highest *H*_E_ was in GXF (0.277), followed by GXG1 (0.275), and the lowest was in GXG3 (0.211). The size ranking of the π in each population was consistent with the *H*_E_ (Table 1 and Figure 1b). The *F*_IS_ coefficient was used to assess the inbreeding level within the populations. The results demonstrated that three wild populations (MZT, SFS, and MJG) and two ex situ populations (GXG1 and GXF) exhibited *F*_IS_ coefficients greater than 0, suggesting the possibility of inbreeding. In addition, all populations had positive Tajima'D values, suggesting that *C. tunghinensis* may have experienced population shrinkage or equilibrium selection. The LD analysis showed that GXF, GXG1, and the four wild populations had comparable LD decay (Figure 1c). However, the GXG2, GXG3, and GXN populations showed slightly slower LD decay than the other populations, suggesting that these ex situ populations may have been subject to stronger selection (Figure 1c).

### 3.3 Population structure

**FIGURE 2:**
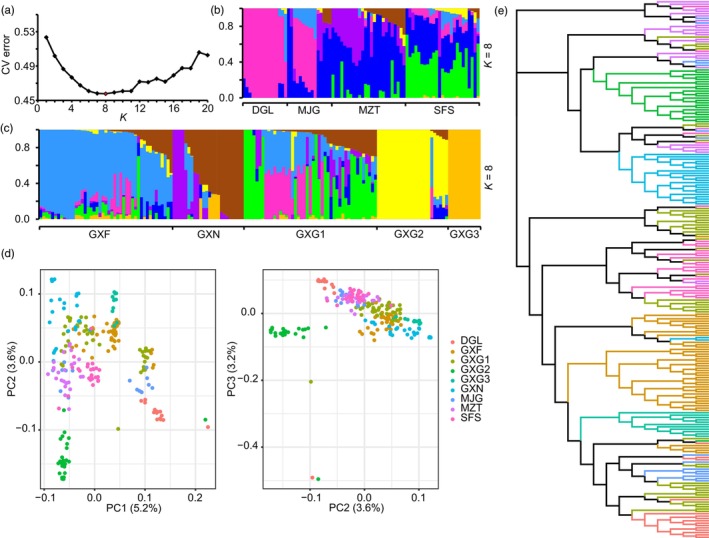
Population structure of *C. tunghinensis*. (a) Cross‐validation (CV) error distribution for *K* from 1 to 20, with *K* of the smallest CV value marked in red. Population structures of wild (b) and ex situ (c) populations, with different colors representing putative ancestral components (*K* = 8). (d) Principal component analysis of 249 individuals, with different colors representing populations. (e) The maximum likelihood phylogenetic tree for 249 individuals. The color of the branches is represented by the legend in (d).

Admixture analysis showed that *K* = 8 was the optimal clustering number among all *C. tunghinensis* individuals (Figure 2a). In the wild populations, most individuals showed evidence of admixture and there were many shared genetic components between populations (Figure 2b). In the ex situ populations, most individuals in GXF, GXN, and GXG1 also demonstrated admixture, although few components were shared between populations, implying possible genetic differentiation between the ex situ populations (Figure 2c). At least five genetic components were detected in the GXG1 population, suggesting that the germplasm origin in the GXG1 population may have been very complex. In contrast, the population structure of GXG2 was single, while that of GXG3 was nearly identical (Figure 2c). The genetic variations explained by the first three principal components (3.2%–5.2%) in the PCA were all low; this was probably because the individuals in the ex situ population were derived from these wild populations, so it is difficult to distinguish between them (Figure 2d). Nevertheless, a few individuals seem to exhibit separation from other individuals, for example, individuals from the GXG2 population, which may be related to the degree of differentiation between this population and other populations. This potential differentiation between the populations was more easily observed in the ML tree; for example, the GXG2 and GXN populations were grouped on different branches from other populations (Figure 2e).

### 3.4 Genetic differentiation and gene flow

**FIGURE 3:**
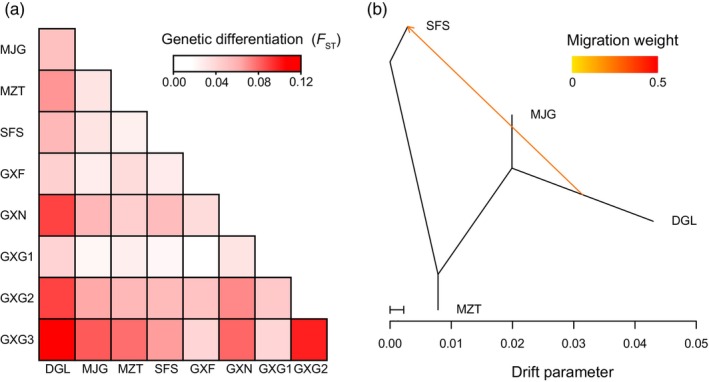
Genetic differentiation and gene flow analysis. (a) Genetic differentiation coefficients (*F*_ST_) for the nine populations. (b) Gene flow analysis of the four wild populations.

The mean *F*_ST_ values for the four wild populations, the five ex situ populations, and between the wild and ex situ populations were 0.044, 0.051, and 0.054, respectively (Figure 3a). The Treemix simulation scenarios (m = 0–5) were evaluated using OptM, and the results showed that the optimal migration model was m = 1. This indicated that a migration event in the past of wild *C. tunghinensis* was the most supported and that it had occurred between DGL and SFS populations (Figure 3b and Figure S2).

### 3.5 Core germplasm extraction

**FIGURE 4:**
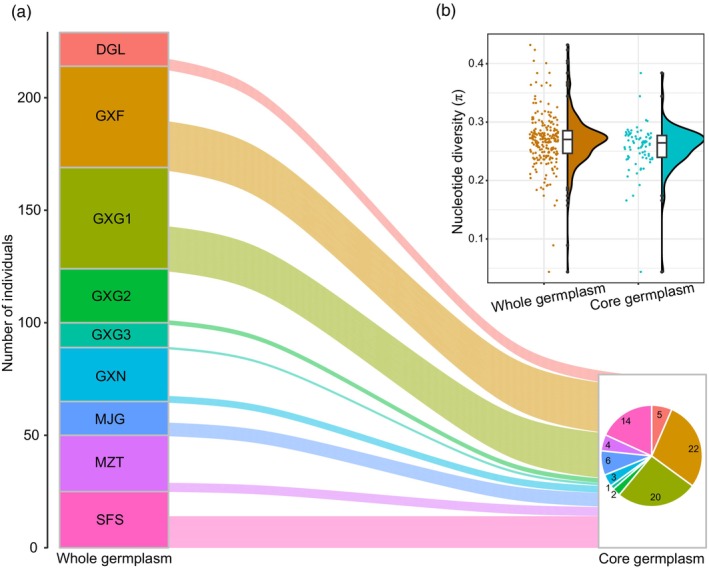
Core germplasm extraction. (a) Seventy‐seven core germplasms were extracted from 229 germplasms. (b) Nucleotide diversities of core and whole germplasm were compared.

The genetic distance matrix between the 229 *C. tunghinensis* germplasm samples was calculated, resulting in the extraction of 77 core germplasms (Table S2). These core germplasms consisted of 22 from the GXF population, 20 from GXG1, and 14 from SFS, among others (Figure 4a). The *H*_E_, *H*_O_, and π values of the core germplasms were 0.281, 0.259, and 0.283, respectively, accounting for 99.29, 95.93, and 99.65% of the whole (Figure 4b and Table S3). These results suggested that to a large extent, the core germplasm could retain the genetic information of the whole germplasm.

## 4 DISCUSSION

### 4.1 High genetic diversity of wild *C. tunghinensis*

The current study is the first to use large‐scale genomic SNP loci to assess the genetic diversity of *C. tunghinensis*. Previously, the genetic diversity of the parental (*H*_O_ = 0.830, *H*_E_ = 0.681) and offspring populations (*H*_O_ = 0.845, *H*_E_ = 0.698) in wild *C. tunghinensis* were calculated based on SSR markers (Tang, Zou, et al., 2020). The values obtained by that study are approximately three times higher than those of the wild populations evaluated here (*H*_O_ = 0.257–0.293, *H*_E_ = 0.247–0.262). Previous investigations have shown that SSR markers estimate heterozygosity two to three times higher than SNP markers (Clugston et al., 2022; Zimmerman et al., 2020). Therefore, the results of the current study are comparable to those obtained by Tang, Zou, et al. (2020). Compared with other endangered shrub species that have been assessed based on genomic SNPs, including *Lonicera oblata* (*H*_E_ = 0.2863, *H*_O_ = 0.1863, π = 0.2874; Mu et al., 2022), *Rhododendron meddianum* (*H*_E_ = 0.0713, *H*_O_ = 0.0505; Zhang et al., 2021), and cultivation type (*H*_O_ = 0.259) and wild type (*H*_O_ = 0.229) *C. sinensis* (Niu et al., 2019), all four wild populations of *C. tunghinensis* exhibited high levels of *H*_E_, *H*_O_, and π (Table 1). These results suggest that despite the small number and fragmented distribution of the populations, wild *C. tunghinensis* maintained a high level of genetic diversity, also consistent with earlier findings (Tang, Zou, et al., 2020). Previous studies have shown that the presence of frequent gene flow between fragmented and small populations is effective in maintaining high genetic diversity (Cai et al., 2020; Hu et al., 2022). Our Admixture results showed that there are many shared genetic components between the wild populations (Figure 2b), which may be genomic evidence for frequent and recent gene flow between populations, as has been observed in previous studies (Ma et al., 2021; Wei et al., 2023). In addition to recent gene flow, we detected an ancient migration event between the wild populations (Figure 4a). These results support a close genetic exchange between wild populations of *C. tunghinensis* over a long period of time, which is the most likely explanation for their current high levels of genetic diversity. In addition, the positive Tajima'D values for both the wild and ex situ populations (Table 1) suggested that they still carried the same genomic traces of past population contraction (Roy et al., 2017). This implies that the currently fragmented and small wild populations may be the result of shrinkage of a larger population. Furthermore, these former larger populations may have created a more favorable environment for the accumulation of genetic diversity.

### 4.2 Evaluation of genetic diversity during ex situ conservation of *C. tunghinensis*

Genetic diversity in small and isolated populations is susceptible to changes due to genetic drift, inbreeding, and selection (Su, Richardson, et al., 2017; Zhao et al., 2018). Most of the ex situ populations met the requirements of small and isolated populations. Previously, the genetic diversity of ex situ populations was found to be lower than that of most wild populations in *C. nitidissima*, a closely related species of *C. tunghinensis* (Wei et al., 2005, 2008). Similar cases have been recently found in *Cycas calcicola* in Australia (Clugston et al., 2022) and *Attalea crassispatha* in Haiti (Diaz‐Martin et al., 2023). Furthermore, Huang et al. (2022) reported that the average population genetic diversity of cultivated *Angelica dahurica* was reduced by about one‐third compared with wild germplasm resources. However, the opposite trend has been detected in some species, including *Amorphophallus albus* (Tang, Liu, et al., 2020) and *Cupressus chengiana* (Chang et al., 2022). These results are in line with those of the current study, suggesting that ex situ populations do not always adequately represent the genetic diversity of wild populations. This representation may depend on differences in the materials used to establish the ex situ populations. For example, establishing *Cupressus chengiana* ex situ populations by the direct transplantation of seedlings from the wild can better preserve the genetic diversity of the wild populations (Chang et al., 2022). In contrast, ex situ populations of *C. nitidissima* established by seed collection were found to have lost some degree of their genetic diversity (Wei et al., 2005, 2008). In reality, preference is most often given to better growth and higher quality seed material for establishing populations in ex situ conservation programs (Havens et al., 2004). Furthermore, the plants in ex situ populations are also inevitably subjected to environmental stress due to possible differences between the ex situ conservation environment and the native habitat. Therefore, successfully established ex situ populations are usually subjected to strong selective effects, a factor that may have a critical influence on their genetic diversity in the short term (Ensslin et al., 2015). Although genetic drift and inbreeding can also have significant effects on genetic diversity in small and isolated populations, these effects may take multiple generations to accumulate (Burgess et al., 2007).

Both the nucleotide diversity and expected heterozygosity in the two ex situ populations (GXF and GXG1) were higher than those in the wild populations (Table 1), which may be attributed to the direct transplantation of wild seedlings as this type of material allows the preservation of genetic diversity from different maternal lines (Griffith et al., 2021). In terms of genetic structure (Figure 2c), the GXG1 population was found to have the most complex origin of the ex situ populations which may have come from multiple different maternal lineages. Although the GXN population was also derived from wild seedlings, it retained significantly fewer adult plants than the GXF and GXG1 populations (Table 1). A meta‐analysis of genetic representativeness in ex situ conservation plants and their wild source populations throughout the world showed that it was difficult to maintain high genetic representativeness when the number of plant samples in the ex situ population was less than 30 (Wei & Jiang, 2021). To the best of our knowledge, all three of the public organizations collected numerous plants and took great care of them when the ex situ populations of *C. tunghinensis* were established, so the reduced plant numbers in these populations may be the result of adaptation to local environmental conditions. LD analysis can reflect the intensity of selection on a population, and slower LD decay usually implies greater selection in the population (Luo et al., 2022; Zhao et al., 2022), confirmed by the apparently higher selection intensities observed in several ex situ populations (Figure 1c). The lower genetic diversity in the GXG2 and GXG3 populations compared with the wild populations is expected and is most likely explained by the fact that they were established from the seeds or cuttings of a small number of elite trees. We conclude that although ex situ conservation by the propagation of seeds or cuttings from elite trees has the potential to provide plant material adapted to the local environment, it may be difficult to capture the genetic diversity of the source populations, and thus, this approach should be used with caution for ex situ conservation programs in the absence of genetic diversity surveys.

### 4.3 Comparison of population structure and genetic differentiation between wild and ex situ populations

An understanding of population structure is vital for identifying the correct conservation and management strategies (Pan et al., 2022; Tong et al., 2020). Admixture analysis showed that although multiple genetic components were detected in *C. tunghinensis*, the structure of the ex situ populations derived from seeds and cuttings was relatively simple compared with the ex situ populations derived from transplanted wild seedlings and wild populations (Figure 2b,c). We further evaluated differentiation between these populations. Typically, *F*_ST_ values in the range of 0.005–0.05 and 0.05–0.15 are indicative of small and moderate differentiation, respectively (Wright, 1978). Using this criterion, *C. tunghinensis* showed small differentiation between the wild populations (mean *F*_ST_ = 0.044) and marginally moderate differentiation between the ex situ populations (mean *F*_ST_ = 0.051). Wild *C. tunghinensis* is found naturally in four populations within a distribution area of less than 100 hm^2^. These trees show limited dispersal distances with seedlings primarily growing around the mother tree, mostly due to the large fruit size. However, the geographical distance between the wild populations was relatively close and the pollinators (primarily sunbirds and bees, personal observation) of golden camellia species could easily accomplish cross‐population pollination, which is also consistent with the Admixture and Treemix inferences. Therefore, the low degree of differentiation seen between the wild populations was not unexpected. In contrast, there were no pathways for gene flow between the ex situ populations, and at the same time, they needed to adapt to the local ex situ environment. Differentiation between the ex situ populations and between ex situ and wild populations would be expected to increase with time (Wei & Jiang, 2021). It is to be noted that when such differentiation reaches a certain threshold, it may lead to a loss of adaptation of these ex situ germplasms to their native habitat, thus limiting the implementation of the more important reintroduction that follows the ex situ conservation program (Enßlin et al., 2011). To avoid these potential risks, it may be necessary to facilitate gene flow between ex situ populations via anthropogenic means, including artificial pollination between ex situ populations or between ex situ and wild populations, as well as systematically supplementing the current ex situ populations with new wild germplasm resources. Especially in consideration of the long‐term and frequent gene flow observed between the wild *C. tunghinensis* populations, there are artificial barriers between the ex situ populations and it is thus necessary to strengthen cooperative breeding programs between different public organizations. However, it is still unclear whether blind crosses between plants from different populations will result in inbreeding or outbreeding depression (Lin et al., 2021; Ma et al., 2022). To investigate this issue, it will be necessary to further evaluate the fitness of progeny obtained from artificially designed inbreeding and outbreeding and to identify suitable cross pairs from the cross trials. The genomic data provided in this study could be used to accurately assess the kinship between these *C. tunghinensis* plants and provide useful support for subsequent inbreeding and outbreeding trials.

### 4.4 Application of core germplasm and recommendations in ex situ conservation

The construction of core germplasm can significantly improve the efficiency of the management and utilization of germplasm, thus reducing costs. This concept is heavily applied in plant resource collection and utilization (Miao et al., 2016), and although it has not been used to a significant extent in this context, it could be further combined with ex situ conservation programs for endangered plants. As the biodiversity crisis intensifies and increasing numbers of plants face threats to their survival, ex situ programs are increasingly used for the conservation of endangered plants (Xu, 2017). Meanwhile, the decreasing cost of high‐throughput sequencing has facilitated the direct assessment of the genetic diversity of germplasm through sequencing (Brhane et al., 2022; Nagano et al., 2022), effectively reducing the risk of genetic diversity loss following the costly establishment of ex situ populations. The identification of core germplasm by combining high‐throughput sequencing followed by ex situ conservation is an important aspect of the future conservation of endangered species, especially considering the limitations of the currently available nursery resources.

In this study, 77 core germplasms were extracted from 229 *C. tunghinensis* germplasms. Based on experience in the agricultural field (Li et al., 1999), our core germplasm was found to represent more than 95% of the genetic diversity of the whole germplasms and can thus be considered an “initial collection,” which can provide a reference of genetic variation for future research and utilization. Our 77 core germplasms contain 53 already introduced and 24 to be introduced in the wild, and we plan to collect seeds or cuttings of these core germplasms for further propagation to reconstruct a core ex situ population, thus enhancing conservation. Finally, this study makes the following recommendations on how to capture as much genetic diversity as possible in ex situ conservation programs:

1. In the absence of genetic diversity surveys, priority should be given to the direct transplantation of wild seedlings for introduction and the collection of material from different maternal lines whenever possible. It is important to back up these transplanted wild seedlings so that they can survive well in the ex situ environment (Hoban, 2019), for example, by expanding the copies of alleles through asexual reproduction to maintain long‐term genetic diversity.

2. Based on an in‐depth understanding of the genetic diversity of the populations, both populations and individuals within the populations that need to be prioritized for conservation should be identified, and we strongly recommend the selection of individuals that require ex situ conservation by constructing core germplasms to improve the efficiency of conservation.

3. Genetic diversity should be used as an important indicator for assessing ex situ conservation and continuous attention should be paid to it. For example, after an ex situ population has been successfully established, the dynamics of genetic diversity, especially between generations, should be recorded through genetic monitoring (Diaz‐Martin et al., 2023).

## CONFLICT OF INTEREST STATEMENT

The authors declare that they have no known competing financial interests or personal relationships that could have appeared to influence the work reported in this paper.

## Supporting information


Figures S1 and S2

Table S1

Table S2

Table S3

## Data Availability

Raw GBS data are available at NCBI with the SRA accession number of PRJNA935504.
