# Supplementary material for: Genome‐wide diversity evaluation and core germplasm extraction in ex situ conservation: A case of golden Camellia tunghinensis
Source: Evol Appl. 2023 Aug 19;16(9):1519–30. doi: 10.1111/eva.13584 (PMC10519411; doi:10.1111/eva.13584)
Supplement: Supplementary file 1 — Figures S1 and S2 [file EVA-16-1519-s003.docx]

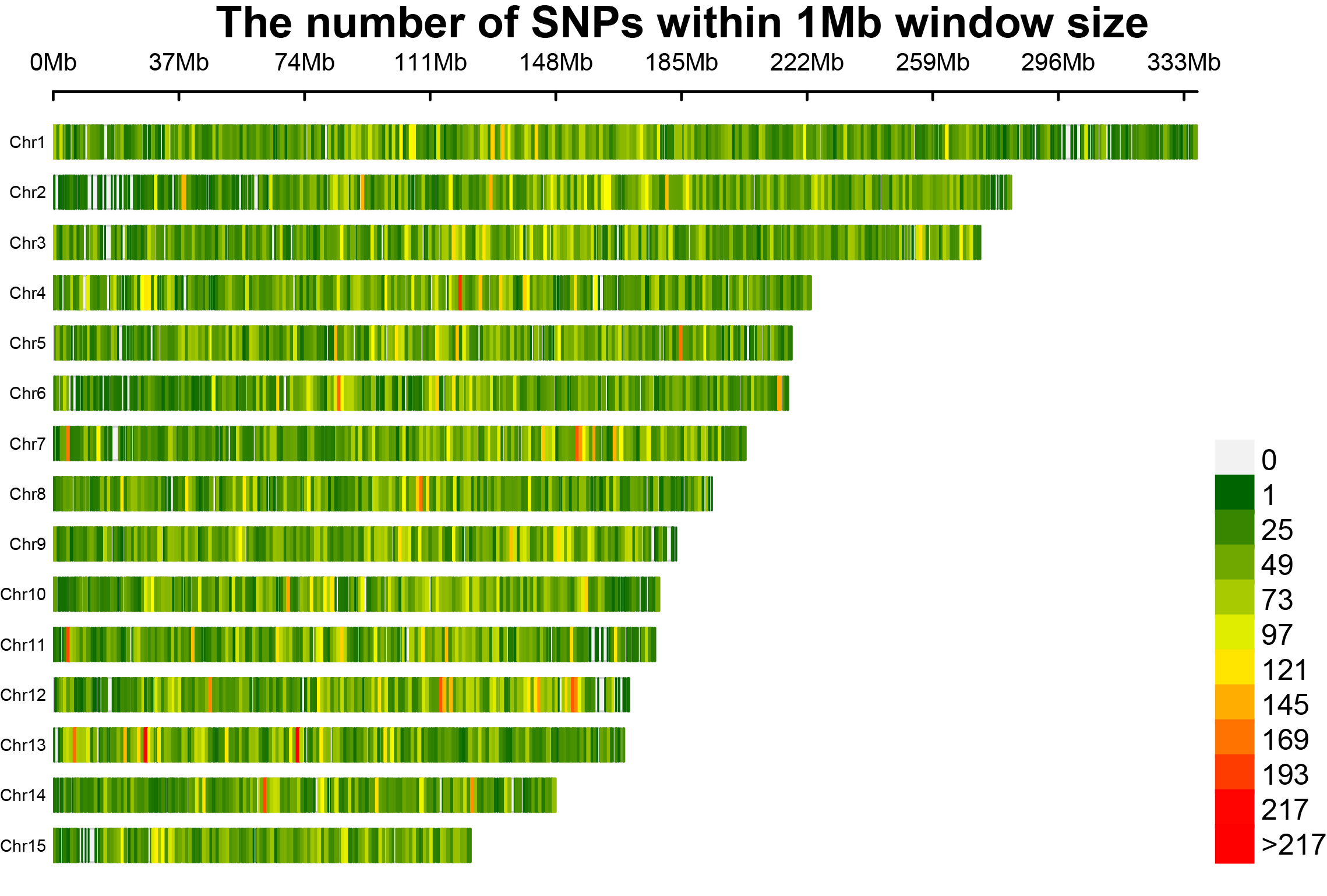


Figure S1 Distribution of SNP loci on the chromosomes of *C. sinensis* reference genome.


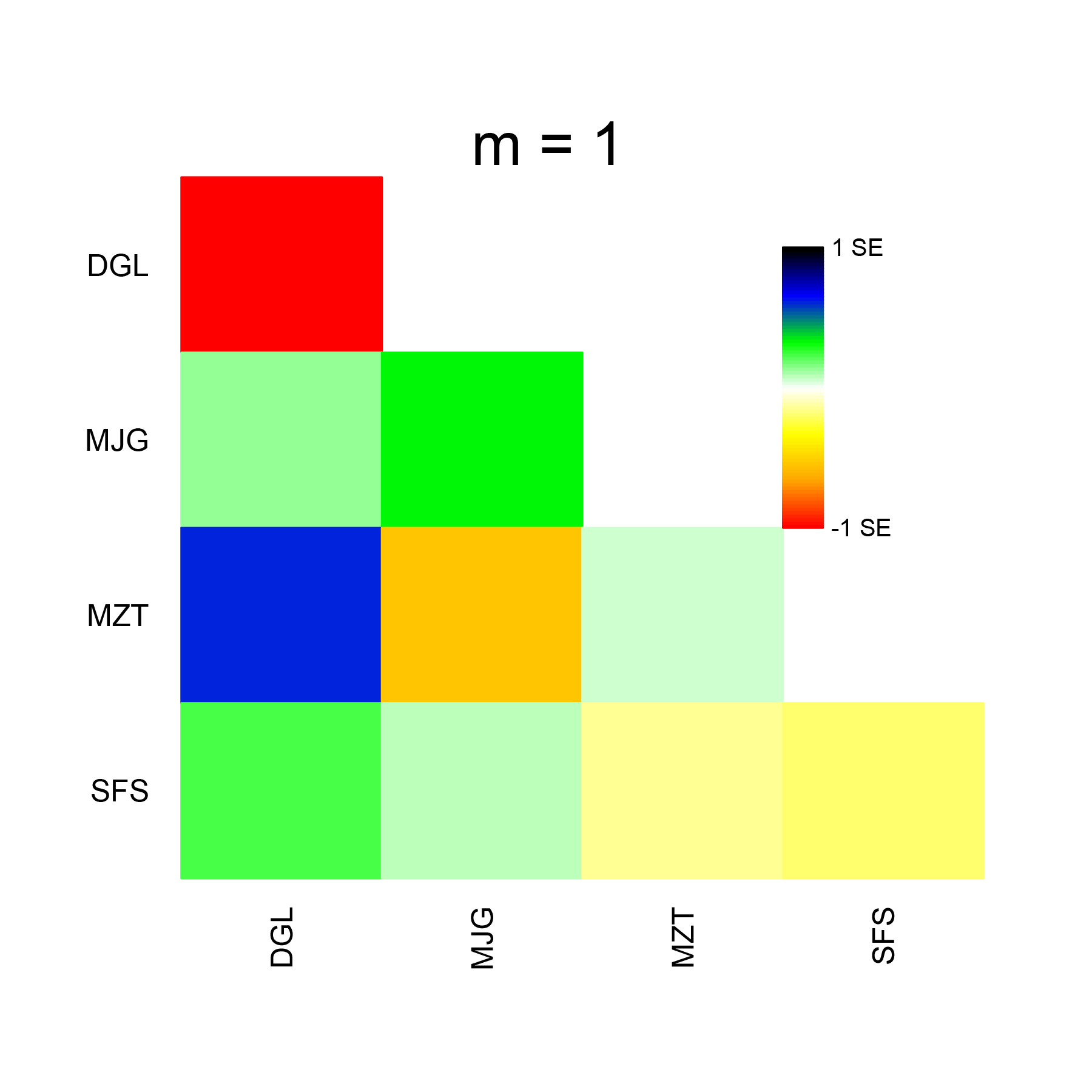


Figure S2 Residuals matrix for migration model (m=1).
